# K‐means cluster analysis of characteristic patterns of allergen in different ages: Real life study

**DOI:** 10.1002/clt2.12281

**Published:** 2023-07-07

**Authors:** Lei Zhao, Jie Fang, Yong Ji, Yingying Zhang, Xin Zhou, Junfeng Yin, Min Zhang, Wuping Bao

**Affiliations:** ^1^ Department of Respiratory Medicine Shanghai General Hospital Shanghai Jiao Tong University School of Medicine Shanghai China; ^2^ Department of Laboratory Medicine Shanghai General Hospital Shanghai Jiao Tong University School of Medicine Shanghai China; ^3^ School of Mathematical Sciences Tongji University Shanghai China

**Keywords:** age, atopy, cluster analysis, eosinophils, immunoglobulin E

## Abstract

**Background:**

Atopy varies in people of different ages owing to different physical conditions and exposure to allergens. We aimed to cluster ages based on atopic severity using K‐means cluster analysis and identify atopic incidence, severity, as well as the association among peripheral eosinophils, IgE and sensitisation.

**Methods:**

Consecutive patients (*n* = 7654) with allergic symptoms and undergoing allergen‐specific IgE tests were included from 2013 to 2017. Age, sex, specific‐IgE, peripheral eosinophil counts and total‐IgE were collected.

**Results:**

Five age categories were identified: 1–17, 18–36, 37–52, 53–69 and 70–100 years. The incidences of atopy and poly‐sensitisation decreased with increasing age. Similar trend was observed for aeroallergens, egg and milk but not for peanuts, soy or seafood. Dust mites remain the crucial factor bothering patients with allergic symptoms, especially for children and adolescents. In patients aged <52 years, sensitisation to aeroallergens was more prevalent than food. In group 37–52 years, incidence of females' atopy was higher than that of males. The overlap of atopy, high eosinophils, and high total‐IgE was found in only 19.18% of patients. The trend of allergen‐test positivity is not parallel to total IgE and peripheral eosinophil counts.

**Conclusion:**

Age‐grouping based on cluster analysis helps to find the changes in atopic status and distribution of sensitised allergens with age. Allergen tests are still necessary in the clinical diagnosis and treatment. An innovative exploration of the influence of age and allergens on total‐IgE and eosinophil counts is helpful for the development of bio‐targeted precision therapy.

**Clinical Trial Registration:**

ChiCTR2300067700.

## 1 INTRODUCTION

In the last two decades, the incidence of allergic diseases, such as asthma, rhinitis and dermatitis, has increased rapidly worldwide, including in China. Data from a random young population showed that approximately 50% of atopic patients, defined as a personal and/or familial tendency to produce IgE antibodies in response to ordinary exposure to allergens, have symptoms referable to atopy, including asthma, rhinitis and dermatitis.^1^ Atopy is associated with the development,^2^ severity and clinical control of allergic diseases.^3^, ^4^, ^5^, ^6^

The incidence of atopy in random populations, defined as the presence of positive(s) on prick skin testing with a small battery of common relevant allergens, ranges from 30% to 50%.^1^, ^7^, ^8^ Age is responsible for the different incidences of sensitisation and clinical reactivity to allergens or its absence in sensitised people.^9^ However, in previous atopy‐related studies, age grouping was often subjective. The participants were conventionally divided into four groups: children (age: 0–12 years), adolescents (age: 13–18 years), adults (age: 19–60 years) and the elderly (age: ≥61 years). This age grouping criterion was not based on epidemiological characteristics of atopy. Other sequential age categories were also used^10^, ^11^, ^12^; however, the reasons and principles of age division were not clarified. In adults aged 19–60 years, further subcategory recognition of age has not been adequately studied, although the heterogeneity of atopic status between ages has been reported.^10^ Inappropriate age classification understates or overinterprets its true impact on atopy and allergen distribution. Therefore, studies on recognition of age category clustering and further analysis to investigate the characteristics of allergen sensitisation in the identified sequential age categories are necessary.

In primary hospitals, total IgE (t‐IgE) and blood eosinophils are commonly used as biomarkers for atopy or allergy. However, their predictive value for atopy requires further exploration. Therefore, a single‐centre retrospective study was performed in consecutive patients who visited the Shanghai General Hospital and their serum allergen specific IgE (s‐IgE) was assessed from January 2013 to December 2017. The aims of this study were to (1) cluster ages based on atopic severity with the K‐means cluster analysis and (2) identify the atopic severities based on the age categories, t‐IgE and peripheral eosinophils in those patients to investigate a possible relationship between these values and atopy.

## 2 METHODS

### 2.1 Study patients

A single‐centre, retrospective study was performed on consecutive patients who visited the Shanghai General Hospital for allergic symptoms and underwent serum allergen S‐IgE tests from January 2013 to December 2017. Patient charts were retrospectively reviewed.

### 2.2 Study design

The age, sex, date of hospital visit and serum allergen s‐IgE test results were collected and analysed. T‐IgE and peripheral eosinophil values were also collected, if available.

The ethics committee of Shanghai General Hospital, Shanghai Jiao Tong University School of Medicine, approved the protocol and a waiver of informed consent was issued for our study (number: 2017KY159).

### 2.3 Allergens test

S‐IgE against 17 allergen extracts and Cross‐reactive carbohydrate determinants were measured using the EUROLINE Atopy (China7) (IgE) Assay Kit, a semi‐quantitative immunoblot method. The 17 allergens could be divided into 10 aeroallergens (willow/aspen/elm, common ragweed, *Artemisia argyri*, dust mites, house dust, cat, dog, *Blattella germanica*, *Penicillium notatum*/branch spore would/*Aspergillus fumigatus*/alternaria, Humulus) and seven food allergens (egg albumen, milk, peanuts, soy, cod/lobster/scallops, shrimp and crab). Atopic severity was stratified into seven grades and assigned numbers 0–6 as the sensitive index (SI) for 0–0.35, 0.35–0.69, 0.70–3.49, 3.5–17.49, 17.5–49.99, 50–99.99 and ≥100 kU/L, respectively; of those, 17 allergens were reviewed. A positive test result was defined as s‐IgE ≥0.35 kU/L. Atopy, categorised as yes or no, was defined as at least one positive test for s‐IgE antibodies. Patients with positive anti‐CCD s‐IgE results were excluded from our analysis.

T‐IgEs were measured using UniCAP 1000 and defined as elevated if they exceeded 60 IU/mL. Eosinophils were measured using an automatic complete blood count with differential blood test and expressed as absolute values and percentages of cell counts. The absolute eosinophil count was considered high when it exceeded 0.15 × 10^9^ cells/L.

### 2.4 K‐means clustering analysis

The K‐means clustering algorithm, a type of unsupervised machine learning used to identify homogeneous subgroups from unlabelled input data,^13^ is used for grouping age into a number of k clusters for allergen sensitisation and minimises the variance of the difference between each cluster and distance in this study.

The analysis was conducted as follows: Step 1: k (number of clusters, starting from 5) data objects were randomly extracted and the centroids of each cluster were computed. Step 2: All data are classified into k clusters by minimising the distance from the centroids of each cluster. Step 3: The centroid of each cluster is recalculated based on the clusters reassigned in Step 2. Steps 2 and 3 are repeated until the data belonging to each cluster converge. Python Anaconda (Python version 3.7, https://www.anaconda.com; Anaconda Inc.) and scikit‐learn 0.24 (sklearn.cluster.KMeans; http://scikit-learn.org/stable/index.html) were used for clustering.

### 2.5 Analysis

Baseline data are presented descriptively. Normality of distribution was assessed using the Shapiro‐Wilk test. Normally distributed data are expressed as mean ± standard deviation. Non‐normally distributed data are expressed as median and IQR. Heatmaps were created using GraphPad Prism (version 9.0; GraphPad Software). Multiple comparisons were made using Dunn's test. Differences between aeroallergens and food allergens were compared using the Mann‐Whitney test. The threshold for statistical significance for all analyses was set at *p* < 0.05.

## 3 RESULTS

### 3.1 Baseline characteristics

A total of 7654 patients (mean ± SD age: 42.33 ± 20.85 years) were included in this study; 3598 (47.01%) were male. The number of people who tested positive for serum s‐IgE was stable between the years (1533, 1321, 1409, 1485 and 1906, respectively, for 2013–2017). The incidence of allergies in each month was roughly stable across years (Figure S1).

T‐IgE was measured in 1863 patients and peripheral eosinophil counts were measured in 5066 patients.

Atopy was confirmed in 4060 (53.04%) patients (Table 1). Each patient was allergic to 2.17 ± 1.59 allergens (SI: 1.766 ± 1.029) (expressed as mean ± SD). In our cohort, 3085 (40.35%) patients had sensitised aeroallergens and 2472 (32.30%) had food allergens. The most common aeroallergens were dust mites, house dust and *B. germanica* and the most common food allergens were peanuts, cod/lobsters/scallops and crabs.

**TABLE 1:** Clinical characteristics of patients sensitised to each allergen.

Allergens	Atopic patients, *n* (%)^a^	Sum of SI	Age, years	Male, *n* (%)^b^	T‐IgE, IU/mL	EOS, %	EOS, ×10^9^/L
Dust mites	1363 (17.81)	3601	33.64 ± 19.80	658 (48.27)	155.4 (44.3–440.3)	3.0 (1.6–6.0)	0.21 (0.11–0.44)
Peanuts	711 (9.29)	879	38.80 ± 21.40	308 (43.32)	84.5 (25.9–230.7)	2.4 (1.3–5.2)	0.18 (0.09–0.35)
House dust	677 (8.85)	943	34.64 ± 20.72	333 (49.19)	162.9 (40.6–439.7)	3.2 (1.5–5.8)	0.22 (0.11–0.11)
Fish combination (cod/lobster/scallops)	582 (7.60)	935	39.51 ± 21.60	297 (51.03)	136.2 (23.0–438.0)	2.7 (1.4–5.1)	0.18 (0.09–0.35)
*Blattella germanica*	559 (7.30)	753	37.26 ± 20.79	273 (48.84)	134.3 (32.2–448.1)	2.7 (1.5–5.7)	0.19 (0.10–0.40)
Crab	548 (7.16)	921	40.15 ± 21.34	287 (52.37)	135.8 (44.7–419.5)	2.7 (1.4–5.2)	0.20 (0.09–0.36)
Egg albumen	499 (6.52)	985	35.23 ± 22.63	266 (53.31)	143.3 (41.2–499.6)	2.6 (1.4–5.1)	0.18 (0.09–0.36)
Shrimp	485 (6.34)	666	42.07 ± 21.28	236 (48.66)	140.7 (35.5–417.5)	2.5 (1.5–4.5)	0.17 (0.10–0.31)
Milk	472 (6.17)	995	34.03 ± 22.06	243 (51.48)	127.8 (42.6–348.7)	3.0 (1.4–5.6)	0.20 (0.10–0.41)
Cat	459 (6.00)	988	32.93 ± 19.39	236 (51.42)	208.2 (41.2–468.3)	3.2 (1.7–6.6)	0.23 (0.11–0.48)
Common ragweed	420 (5.49)	615	36.80 ± 20.98	210 (50.00)	116.3 (28.4–450.0)	2.7 (1.3–5.5)	0.18 (0.09–0.40)
Dog	389 (5.08)	859	36.87 ± 18.95	176 (45.24)	150.5 (57.0–450.9)	3.3 (1.7–6.6)	0.25 (0.12–0.50)
Mould combination	384 (5.02)	487	38.65 ± 20.62	203 (52.86)	96.0 (24.0–444.6)	2.9 (1.5–5.7)	0.20 (0.10–0.39)
*Artemisia argyi*	380 (4.9.6)	576	37.25 ± 21.44	203 (53.42)	82.30 (24.7–332.0)	2.7 (1.4–4.8)	0.17 (0.09–0.35)
Humulus	313 (4.09)	684	34.31 ± 20.35	169 (53.99)	163.1 (61.6–558.6)	3.7 (1.8–6.9)	0.27 (0.11–0.49)
Soy	301 (3.93)	453	40.44 ± 20.81	157 (52.16)	104.4 (30.7–552.8)	2.6 (1.4–5.1)	0.16 (0.10–0.33)
Tree combination	272 (3.55)	368	38.96 ± 20.16	139 (51.10)	126.5 (26.3–525.4)	2.8 (1.2–5.6)	0.19 (0.09–0.36)
Inhalant allergens	3085 (40.35)	9874	36.86 ± 20.62	1490 (48.30)	104.4 (29.9–347.9)	2.8 (1.5–5.6)	0.19 (0.10–0.39)
Non‐inhalant allergens	2472 (32.30)	5834	39.35 ± 21.84	1204 (48.71)	84.35 (25.8–296.9)	2.5 (1.4–5.0)	0.18 (0.09–0.35)
All	4060 (53.04)	15,708	38.81 ± 21.18	1930 (47.54)	82.5 (24.7–266.7)	2.7 (1.4–5.2)	0.18 (0.10–0.37)

Abbreviations: EOS, eosinophil; IgE, immunoglobulin E; SI, severity index; T‐IgE, total IgE.

^a^ Percentages in the whole cohort.

^b^ Percentages in each category.

We investigated the variations in age among patients sensitised to different allergens (Table 1). Patients sensitised to cat (32.93 ± 19.39 years), dust mites (33.64 ± 19.80 years), or milk (34.03 ± 22.06 years) were younger than those sensitised to crab (40.15 ± 21.34 years), soy (40.44 ± 20.81 years) or shrimp (42.07 ± 21.28 years).

Visual exploration of the heatmap data (Figure S2) identified several age groups with a relatively high prevalence of atopy. Therefore, the K‐means cluster analysis was performed to identify homogeneous subgroups based on age for allergen sensitisation.

### 3.2 K‐means cluster analysis

**FIGURE 1:**
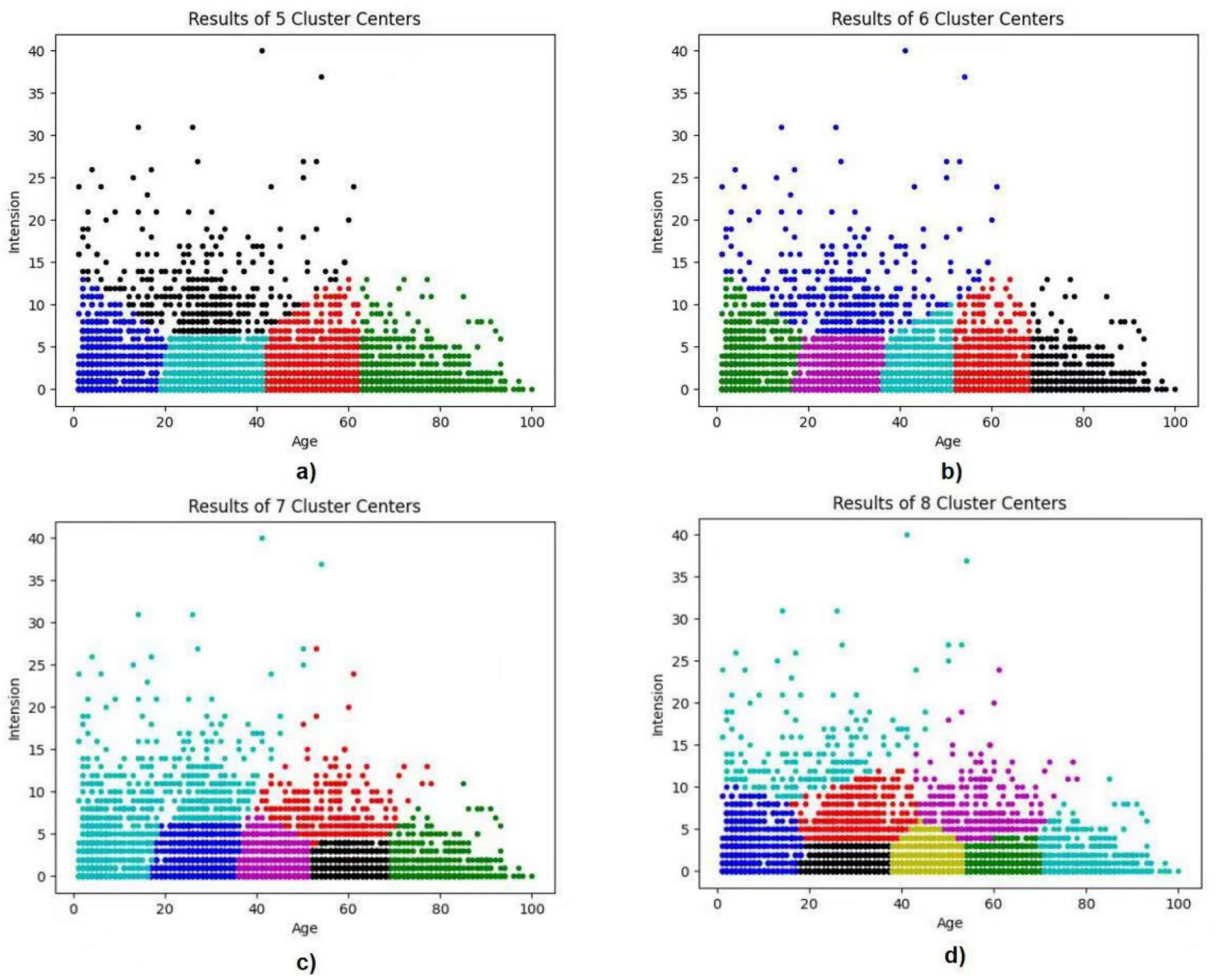
K‐means clustering analysis. The distribution of the patient clusters defined by age and severity index analysed with (A) five cluster centres, (B) six cluster centres, (C) seven cluster centres and (D) eight cluster centres.

We performed the K‐means cluster analysis with five, six, seven and eight centres. The results of five cluster centres classified all patients into four age categories: 1–20, 19–42, 42–62 and 63–100 years (Figure 1), whereas the results of six cluster centres classified all patients into five age categories: 1–18, 17–36, 37–52, 53–69 and 70–100 years. The age grouping of patients was stable and data belonging to each cluster converged when the cluster centres were increased to seven and eight, compared with results of the K‐means cluster analysis with six cluster centres (Figure 1).

Accordingly, the K‐means cluster analysis grouped ages into five clusters for allergen sensitisation: 1–17 (with a centroid of 5), 18–36 (with a centroid of 28), 37–52 (with a centroid of 45), 53–69 (with a centroid of 60) and 70–100 (with a centroid of 78) years.

### 3.3 Distribution of sensitised allergens by five age categories

**FIGURE 2:**
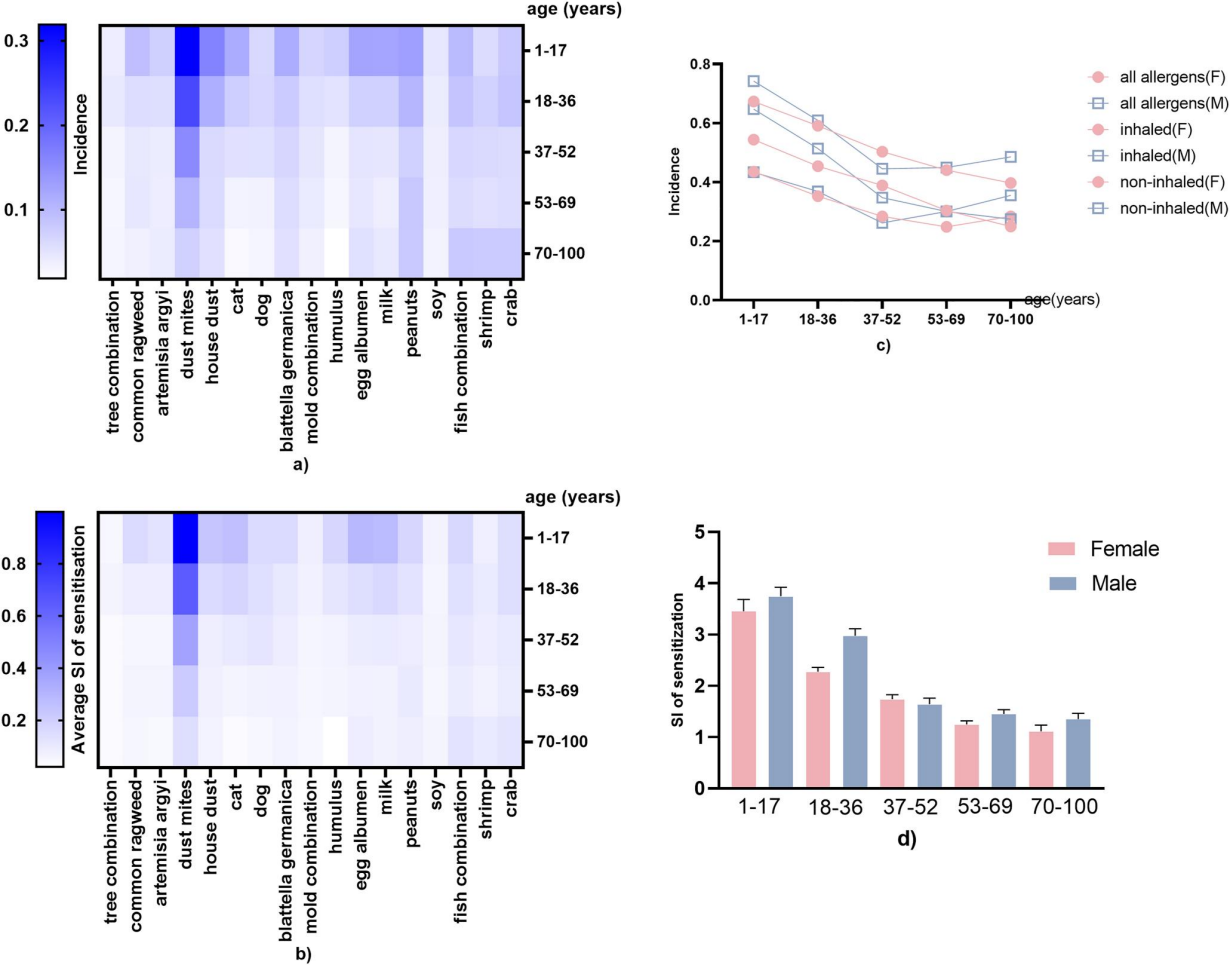
The incidence (A, B) and severity index (B, D) of atopy by the five age categories analysed for different allergens (A, B), aeroallergens and food allergens (C) and different sex (D).

**FIGURE 3:**
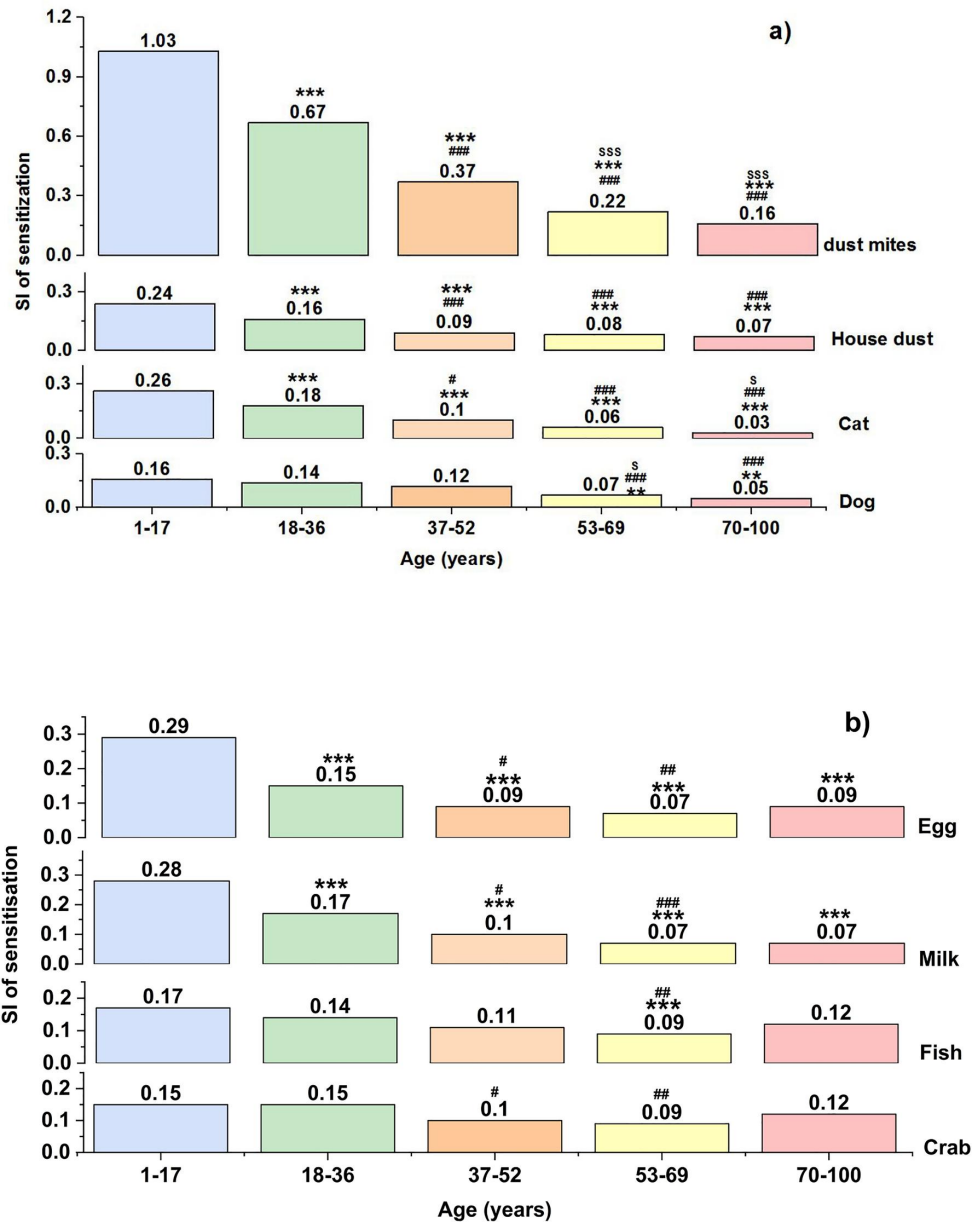
Multiple comparisons of the severity of sensitisation to different allergens by the five age categories. (A) Aeroallergens; (B) food allergens. *, # and $ indicate *P* < 0.05; ** and ## indicate *P* < 0.01; ***, ### and $$$ indicates *P* < 0.001. *compared with the age group of 1–17 years; # compared with the age group of 18–36 years; $ compared with the age group of 37–52 years.

The incidence of atopy decreased with increasing age: 71.41% for the age group of 1–17 years, 59.79% for 18–36 years, 47.60% for 37–52 years, 44.50% for 53–69 years and 37.62% for 70–100 years (Table S1). A similar trend of decreasing incidence with age was observed for the most common aeroallergens: dust mites, house dust, cats, *B. germanica* and mould combination; sensitisation to them had the highest incidence and was the most severe in the age group of 1–17 years (Figures 2A and 3A). The incidence of sensitisation to dust mites, which was the most common allergen, was 32% in the age group of 1–17 years, with an average SI of 1.00.

**FIGURE 4:**
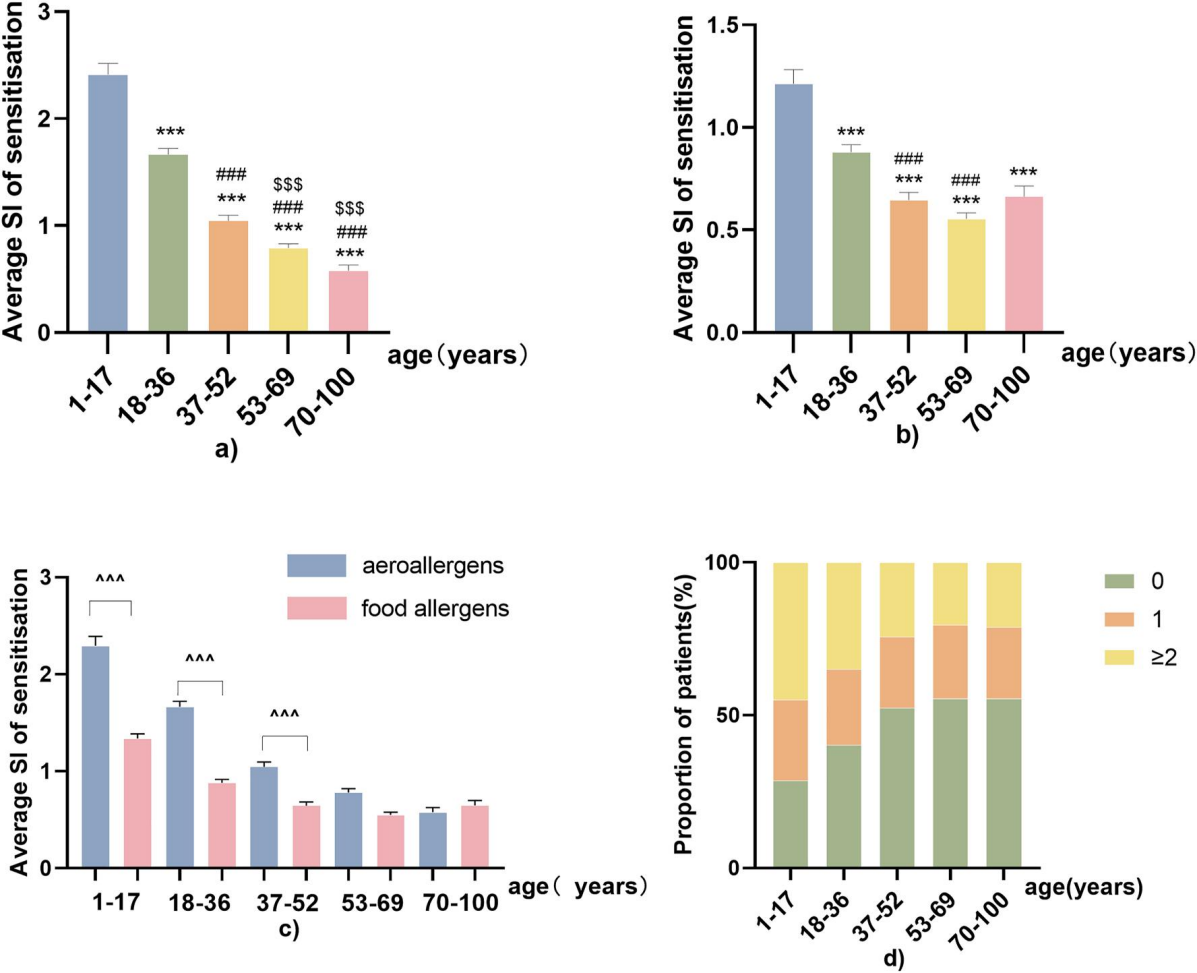
Severities of sensitisation to allergens. Multiple comparisons of different age categories for aeroallergens (A) and food allergens (B); inter‐group comparison between aeroallergens and food allergens by the five age categories (C); and percentage of patients who were nonallergic, allergic to one allergen, or allergic to multiple allergens grouped by the five age categories (D). Data are expressed as mean ± SEM. ***, ###,$$$ and ^^^ indicate *P* < 0.001, *** compared with the age group of 1–18 years, *P* < 0.001; ### compared with the age group of 19–36 years, *P* < 0.001; $$$ compared with the age group of 37–51 years, *P* < 0.001.

However, the incidence of food allergens exhibited different traits. Sensitisation to egg or milk was more prevalent in the age group of 1–17 years, with the highest SI as well (Figures 2A and 3B). Sensitisation to peanuts and soy maintained a stable incidence and severity among the five age categories. Among all of them, only sensitisation to seafood (crab, shrimp and fish combination) had a higher incidence and severity in the age group of 70–100 years compared with the incidence in the age groups of 37–52 and 53–69 years (Figure 3B). In patients aged <52 years, SI of aeroallergens was higher than that of food allergens (Figure 4C). Aeroallergens and food allergens had a similar incidence of sensitisation in patients aged 53–100 years. Only in 37–52 years, the incidence of females was higher than males but males' SI was higher than females in all age groups (Figure 2C,D).

The proportion of patients who were allergic to only one allergen was similar across all age categories (Figure 4D). The incidence of poly‐sensitisation decreased with increasing age. Overall, 45% of adolescents and teenagers were allergic to more than two allergens. Thus, the proportion of non‐atopic patients increased with age.

### 3.4 The characteristics of t‐IgE and eosinophil in atopic patients

Peripheral and t‐IgE and eosinophil counts varied in patients allergic to different allergens (Table 1). Patients sensitised to cats had the highest t‐IgE (208.2 [41.2–468.3] IU/mL) and those sensitised to *A. argyri* had the lowest t‐IgE (82.30 [24.7–332.0] IU/mL). The highest peripheral eosinophil count was found in patients allergic to humulus (0.27 [0.11–0.49] × 10^9^/mL) and the lowest value was found in those allergic to soy (0.16 [0.10–0.33] × 10^9^/mL).

### 3.5 Overlap of atopy, high eosinophil count and high T‐IgE level

**FIGURE 5:**
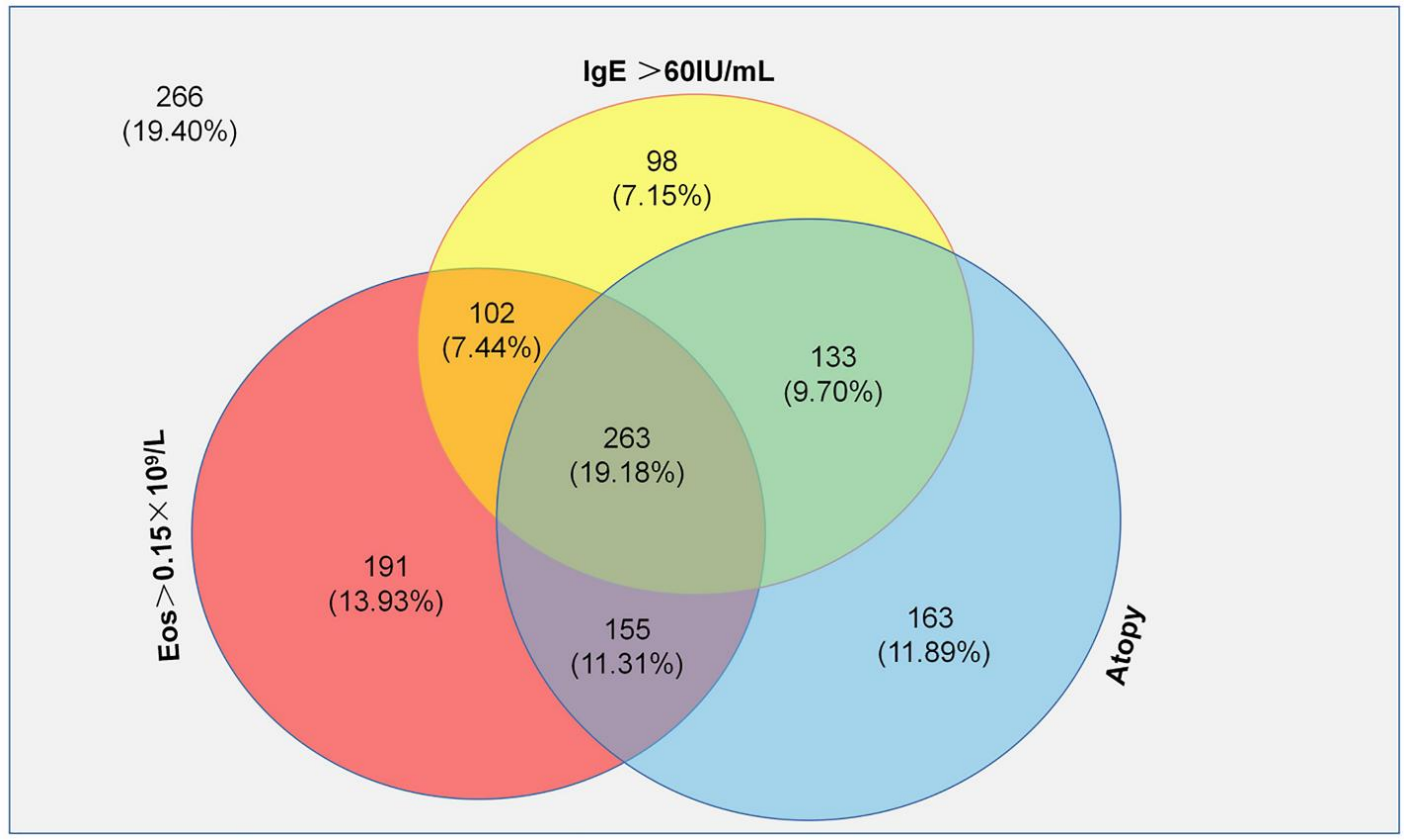
Venn diagram indicating the distribution of total IgE and eosinophils in patients.

A total of 1371 patients provided the results of all three tests (serum S‐IgE, T‐IgE and peripheral eosinophils; Figure 5). Of these, only 263 (19.18%) patients had atopy, high eosinophil counts and high t‐IgE. A total of 266 (19.40%) patients had normal results on all three tests. High eosinophil counts were noted in approximately half of the atopic patients, as were high t‐IgE. One‐fourth of atopic patients had both high eosinophil counts and t‐IgE. However, there were 28.52% of patients without positive allergen test though they had high level IgE or eosinophils.

## 4 DISCUSSION

This study used the K‐means cluster analysis to identify the more natural age categories retrospectively in a consecutive cohort of 7654 patients who underwent allergen testing over a 5‐year period. As we know, this is the first research focusing on the relationship among atopy, t‐IgE and eosinophils in such a large population. We found that SI and proportion of poly‐sensitisation were decreased with age increasing, unrelated to allergen types; SI of aeroallergens was higher than food allergens in every age group; incidence of female's atopy was higher than male's atopy in 37–52 years, but SI of female sensitisation was lower than male in all age groups, based on our age categories. Besides that, the trend of allergen‐test positivity is not parallel to t‐IgE and peripheral eosinophil counts. We believe that our findings will be helpful in the performance of allergic disease research for different age groups.

Immunoblotting test of serum s‐IgE is used to evaluate the sensitisation of various allergens and has high specificity as it avoids the influence of drug combination and operator experience.^14^ High serum s‐IgE predicts more airway events than the skin prick test.^15^, ^16^ Furthermore, the pain and inconvenience of acupuncture have a negative impact on patients' willingness to undergo tests and limit the number of allergen SPTs performed. Immunoblotting test for serum s‐IgE lacks this limitation and could provide complete information about sensitisation to more allergens in this study, as both aeroallergens and food allergens were assessed and collected for each patient.

Results of our K‐means clustering analysis revealed that all aeroallergens, egg and milk showed a descending trend with increasing age, regardless of the incidence or severity of sensitisation, as well as the incidence of allergic poly‐sensitisation.^17^ Therefore, more attention should be paid to sensitisation in children and adolescents. Gender differences in atopy were observed in some age groups. Males' atopy incidence was higher than female in all age groups except in 37–52 years, but as for SI of sensitisation, males may have a higher SI than females in all age groups. Sex hormones may explain sex differences in allergic diseases, which increase the risk of sensitisation in 37–52 years' females.^18^, ^19^, ^20^ Previous studies only indicated that allergic diseases are more common in males than females before puberty, with a reversal after puberty.^21^

Distinct allergens caused different degrees of increasing IgE and eosinophils; cats in our study had the highest level of t‐IgE. A previous study reported that allergens from mammals had more association with high s‐IgE.^22^ The higher level of eosinophils seemed to be distributed in aeroallergens more often. It proved that aeroallergens were capable of driving the increase in SI, IgE and eosinophils more often. However, allergen‐test positivity is not highly parallel to t‐IgE and peripheral eosinophil counts.^10^, ^23^, ^24^ Therefore, the effectiveness of t‐IgE or blood eosinophil count alone in determining atopy is limited and a combination of the two is more valuable in ruling out or predicting allergies. Whether bio‐therapy is needed for patients with a high level of IgE or eosinophils deserves to be explored, although there has a result of negative allergen test.

Dust mites are a major source of perennial allergens and a significant trigger for asthma and allergic rhinitis.^2^, ^25^, ^26^, ^27^ An epidemiological survey identified that dust mites are the most common allergens in south China^28^, ^29^ and are affected by the natural environment and climate, including temperature and humidity,^30^ consistent with our findings. In our study, the incidence and SI of dust mite sensitisation were the highest among all allergens in patients aged 1–52 years. For patients aged >53 years, the SI of sensitisation decreased with age and there was no significant difference in the severity of sensitisation between patients aged 53–69 and 70–100 years. Mite microhabitats within the domestic environment are an ecological stratum for allergen exposure and provide possible opportunities for intervention measures to reduce allergen load. However, the clinical benefits of mite avoidance remain unclear. A meta‐analysis of 44 trials on mite avoidance concluded that mite control measures are not recommended for asthma.^31^ But their conclusions were challenged because they did not distinguish between adult and paediatric studies.^32^ The tendency of dust mite sensitisation to decrease with increasing age noted in our study suggests possible benefits of mite avoidance in children. For children and adolescents, early allergen‐control intervention may be effective in reducing the number of sensitised children with asthma attending the hospital with asthma exacerbations in adulthood.^33^, ^34^, ^35^

As for food allergens, the two most common food allergens are milk and eggs. Sensitisation to eggs and milk is more prevalent in children than in adolescents and adults,^36^, ^37^, ^38^ consistent with our findings. In contrast, seafood allergy affects a substantial proportion of adults, many of whom develop the disease during adulthood.^39^ The mechanism remains unknown, perhaps because of the degeneration of the immune system and gastrointestinal tract function,^40^ which leads to a slight increase in the severity in patients aged 70–100 years. The incidence of peanut allergy was maintained at a stable low severity among all groups, indicating that peanut allergies may persist throughout life.^41^, ^42^

Our study has some limitations. First, our data were obtained from a single centre, although some of our patients came from adjacent provinces such as Jiangsu, Anhui and Zhejiang, which can reflect the condition of east China. Therefore, large‐scale epidemiological studies need to be performed. In addition, the statistical methods used in the analyses assume a cause‐effect relationship between the risk factors and atopy. Future prospective intervention studies are necessary to explore the mechanisms of allergy in specific age groups.

## 5 CONCLUSION

Distinct demographic features such as age are associated with atopy and allergic diseases. In our large‐scale cohort study, the sensitive indexes of most allergens decreased with increasing age. Increased seafood sensitisation was noted in the elderly. Additionally, an innovative exploration of the influence of age and allergens on t‐IgE and eosinophil counts is helpful for the determination of bio‐targeted precision therapy. The inconsistency of changes on atopy, t‐IgE and eosinophil was shown for the first time. Allergen tests are still necessary in the clinical diagnosis and treatment. The combination of IgE and eosinophil counts should be considered when ruling out or predicting atopy.

## AUTHOR CONTRIBUTIONS

**Lei Zhao**: Formal analysis (lead); investigation (lead); methodology (equal); writing – original draft (lead). **Jie Fang**: Methodology (lead). **Yong Ji**: Writing – original draft (equal). **Yingying Zhang**: Investigation (equal). **Xin Zhou**: Conceptualization (equal); validation (equal). **Junfeng Yin**: Formal analysis (supporting); methodology (equal). **Min Zhang**: Conceptualization (lead); methodology (supporting); writing – original draft (supporting), **Wuping Bao**: Conceptualization (lead); investigation (lead); methodology (lead); writing – original draft (supporting).

## CONFLICT OF INTEREST STATEMENT

The authors declare that they have no relevant conflicts of interest.

## Supporting information

Supporting Information S1

## Data Availability

The data that support the findings of this study are available on request from the corresponding author. The data are not publicly available due to privacy or ethical restrictions.
