# Supplementary material for: K‐means cluster analysis of characteristic patterns of allergen in different ages: Real life study
Source: Clin Transl Allergy. 2023 Jul 7;13(7):e12281. doi: 10.1002/clt2.12281 (PMC10326892; doi:10.1002/clt2.12281)
Supplement: Supplementary file 1 — Supporting Information S1 [file CLT2-13-e12281-s001.docx]

**Online Supplement**

**eTable 1 Clinical characteristics of patients grouped by five age categories.**

| **Age categories (years)** | **1–17** | **18–36** | **37–52** | **53–69** | **70–100** |
| --- | --- | --- | --- | --- | --- |
| For the whole cohort, n=7654 | | | | | |
| n (percentage in the whole cohort) | 962 (12.57) | 2241 (29.28) | 1687 (22.04) | 2126 (27.78) | 638 (8.34) |
| Centroid, years | 5 | 28 | 45 | 60 | 78 |
| Male, n (percentages in each category) | 567 (58.94) | 860 (38.38) | 797 (47.24) | 1028 (48.35) | 346 (54.23) |
| Atopic incidence in each category, % | 71.41 | 59.79 | 47.60 | 44.50 | 37.62 |
| Atopic patients, n=4060 | | | | | |
| Sensitive SI of each category | 4 (2–7) | 3 (2–6) | 3 (1–5) | 2 (1–4) | 2 (1–4) |

Abbreviations: SI, severity index


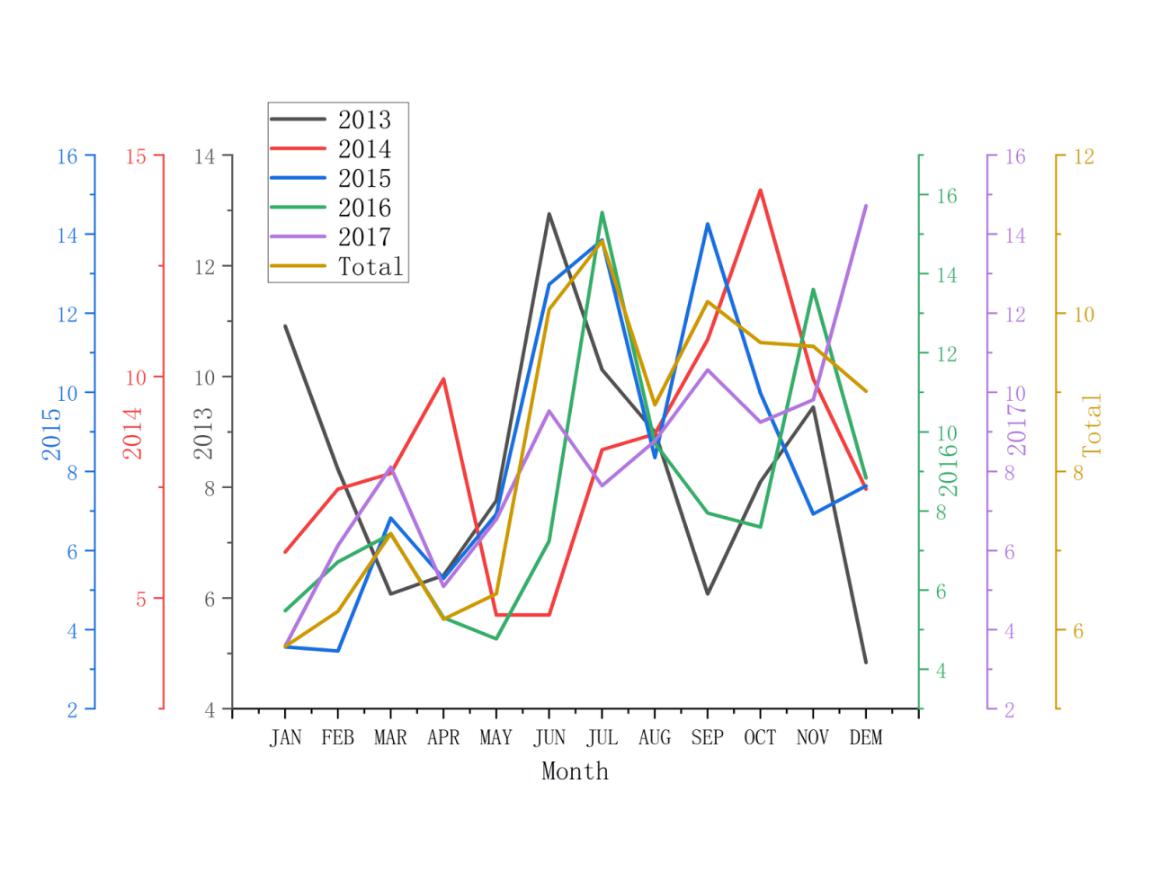


**eFigure 1** Multiple Y-axis line chart showing the similarity of sensitisation patterns for every allergen every year. The Y-axis represents the total level of sensitisation to certain allergens.

**
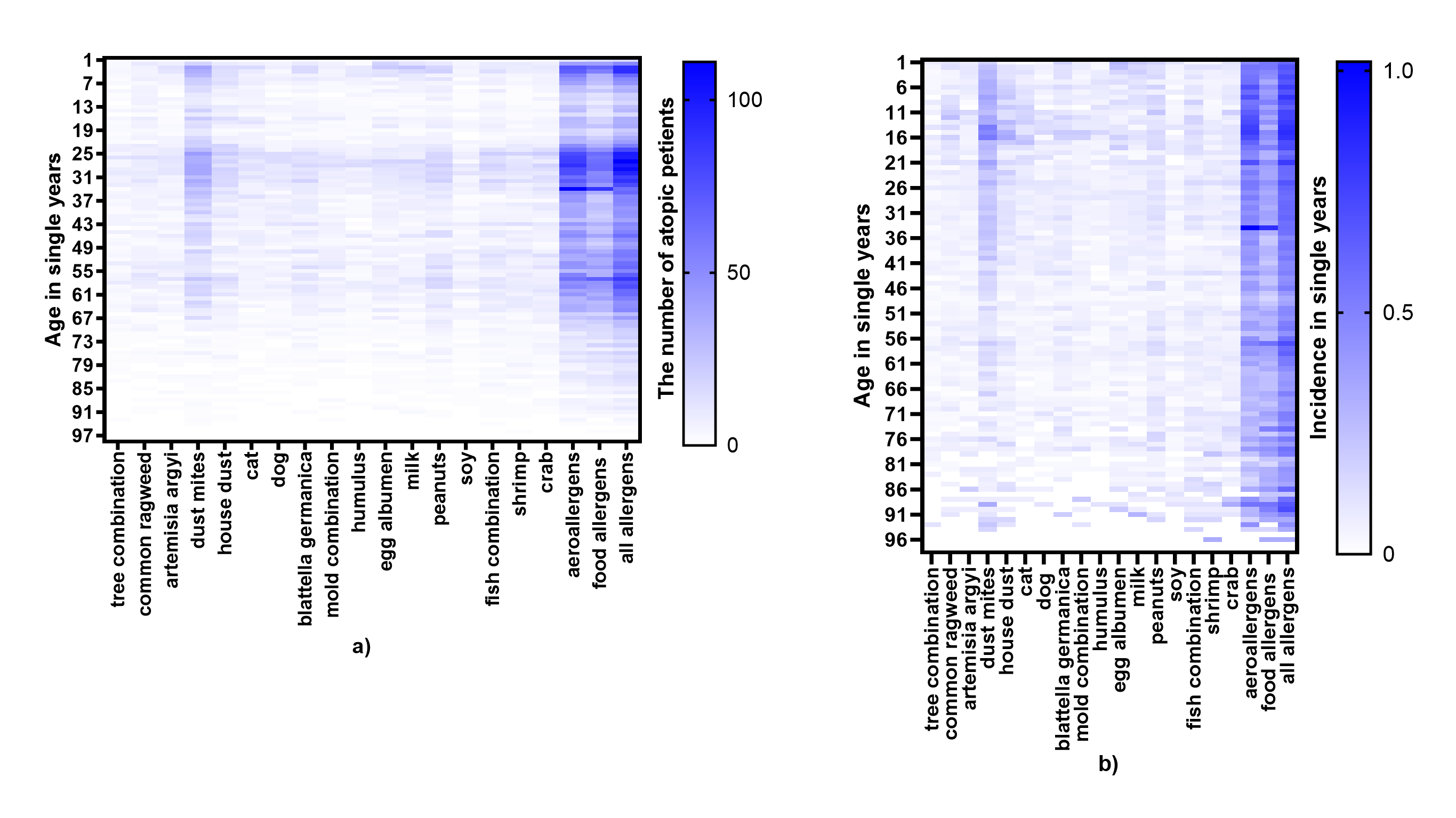
**

**eFigure 2.** Patient number (a) and incidence (b) of sensitization to different allergens for each single age from 0 to 100 years.
